# Science, art, society and Klimt’s University of Vienna paintings

**DOI:** 10.7554/eLife.50016

**Published:** 2019-08-01

**Authors:** Alberto E Pereda

**Affiliations:** Dominick P Purpura Department of NeuroscienceAlbert Einstein College of MedicineNew YorkUnited States

**Keywords:** art and science, science and society, history of science, Gustav Klimt

## Abstract

At the turn of the 19th century the Austrian artist Gustav Klimt was commissioned to decorate the ceiling of the Great Hall of the University of Vienna. However the three paintings he produced – *Philosophy*, *Medicine* and *Jurisprudence* – were rejected by the university and later destroyed by retreating German troops during World War II. The story of these paintings, and another called *Goldfish*, illuminates common ground between art and science, and highlights ongoing tensions in the relationships between art, science and society.

The city of Vienna underwent a major transformation during the second half of the 19th century. The centerpiece of this transformation was the replacement of the traditional walls of the city by a wide avenue, the Ringestrasse, which provided space for the construction of a number of new public buildings (Rentetzi, 2004).

The modernization of the city’s architectural landscape was mirrored by developments in the arts and sciences, which reached new levels of excellence during this period. The Vienna Secession movement, led by Gustav Klimt among others, involved artists breaking away from mainstream art organizations and government control to explore the modernist ideas that were emerging in Berlin, Munich and other European cities.

In the sciences, the Second Vienna Medical School led by Carl von Rokitansky put Vienna at the center of modern western medicine. Rokitansky introduced more scientific approaches to medicine and was a pioneer in the field of pathology. Other prominent physicians in Vienna around this time included the surgeon Theodor Billroth, the clinician Josef Skoda, and the anatomist Josef Hyrtl. Another recruit was Ernst von Brücke, the German physiologist who (with Emil Du Bois-Reymond, Carl Ludwig and Hermann von Helmholtz) argued that all physiological processes can be explained by underlying physical or chemical mechanisms, thus countering the theory of 'vitalism' that prevailed at the time (White, 2006).

Brücke went on to have a prominent role in the scientific development of the university. Among those he trained were Ludwig Mauthner, whose description of the nerve cells in fish (Seyfarth and Zottoli, 1991) has been central to my own research for the last thirty years, and Sigmund Freud, the founder of psychoanalysis. The influence of Brücke and Helmholtz led Freud to conceive of the human mind as a stream of psychological 'energy', or libido, which is continuously transformed (as in Helmholtz’s law of the conservation of energy) into thoughts and behaviors.

Interactions between scientists and artists were common in the intellectually curious Vienna, and modernist ideas in art and science periodically clashed with the traditionally conservative values of Viennese society (Kandel, 2012). Brücke’s materialistic views on science were opposed by Hyrtl, who favored traditional philosophical and religious dogma on science and medicine (Seebacher, 2006). Tensions within the world of art were less evident as the Secession movement, although independent, was sponsored by the state. However, as we shall see, the situation changed when the University of Vienna commissioned Klimt to produce a set of paintings.

## The University of Vienna ceiling paintings

Gustav Klimt was born in 1862 into a family of gold engravers and dedicated his life to art. In 1894, already well-known for his work on buildings on the Ringestrasse, Klimt and his associate Franz Matsch were invited to paint five panels for the ceiling of the Great Hall of the University of Vienna. The theme of the panels was to be *The triumph of light over darkness*: Matsch was to paint the central panel and a panel called *Theology*, whereas Klimt was to paint *Philosophy*, *Medicine*, and *Jurisprudence* (images of all the panels can be seen at this Wikipedia page).

Klimt's first contribution, *Philosophy*, was exhibited for the first time in 1900, and while it was acclaimed in France (it won the Grand Prix at the Paris World Fair), it provoked a strong adverse reaction from university and state officials in Vienna. The reason: Klimt broke with the expected, previous classicist style of his paintings and entered into a new style in which nudity and non-explicit symbolism contrasted with the rationalistic statements of classicist art. Rather than being a celebration of the cultural place that Vienna then had in the world, the painting portrayed a passive, instinctual, interpretation of philosophy. The instinctual insights of modernist art resonated with novel scientific views of the human psyche developed by Freud, who postulated that the majority of our mental life is unconscious and that our civilized life is driven by instinctual bouts of eroticism and aggression that surface to consciousness in the form of words and shapes (Kandel, 2012). Coincident with *Philosophy*, Freud's ground-breaking book *The Interpretations of Dreams* was published in 1900 (Freud, 1900).

**Figure 1.**
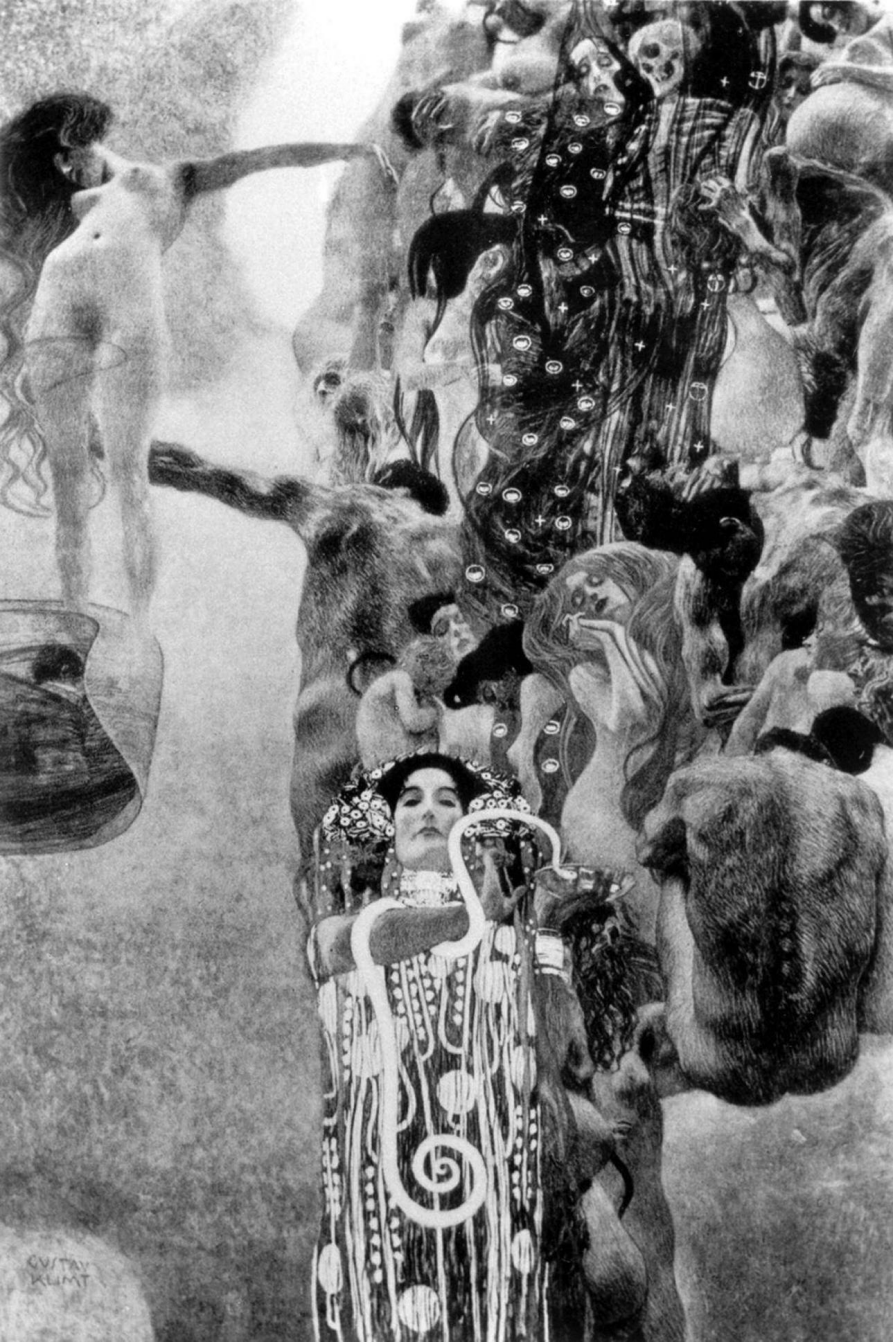
Medicine by Gustav Klimt. This painting was commissioned for the Great Hall of the University of Vienna. It was exhibited for the first time at the tenth Secession exhibition in 1901 and was destroyed in 1945.

While still embattled in the bitter debate triggered by *Philosophy*, Klimt unveiled a second painting, *Medicine*, in 1901. Continuing with his new instinctual style, it depicts a column of nude bodies in which the presence of a pregnant woman, babies and skeletons allude to the unity of life and death (Figure 1). The nude body of an unconscious young woman seems to drift away, held in place by the strong arm of a man. This seems to suggest that no one can escape from the stream of life. At the bottom of the painting, the figure of Hygia, daughter of the god of medicine Asclepious and the goddess of healing, holds the cup of Lethe and the Aesculapian snake while, self-absorbed, turning her back on human suffering. The significance of the images contained in this stunning painting have been the subject of many essays and interpretations (see, for example, Finn et al., 2013; Kandel, 2012; Sark, 2011; Schorske, 1981). There is, however, an obvious message: we are born to die, and medicine – represented by the impotent Hygia – cannot alter our fate.

CREDIT: Art Collection 3/Alamy Stock Photo.

*Medicine* triggered an even yet stronger reaction than *Philosophy*. In addition to featuring explicit female sexuality, which was considered deeply offensive at that time, the painting was not what medical doctors and administrators were expecting. Rather than celebrating Vienna’s position in the world of medicine, Klimt's painting exposed – using an instinctual language – the limitations of our ability to heal and the inevitability of death. In other words, the true nature of medicine was in question. The painting was attacked by a medical journal, and a group of university faculty registered a formal complaint with the university (Bitsori and Galanakis, 2002).

**Figure 2.**
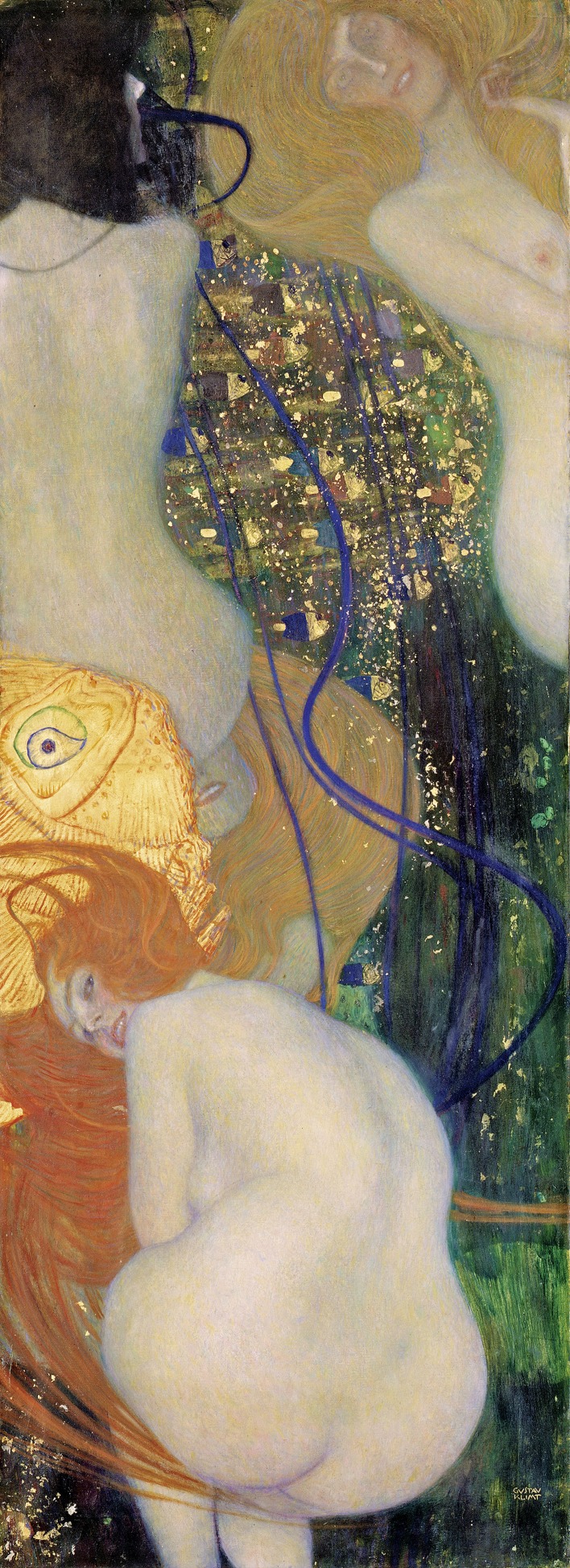
Goldfish, 1901–1902 by Gustav Klimt. Originally entitled *To my detractors*, *Goldfish* reflects Klimt’s response to the criticisms he received for *Philosophy* and *Medicine*.

There were drastic consequences for Klimt after this second scandal: his professorship at the Academy of Fine Arts was not renewed and the Vienna Secession movement lost the support of the state. Klimt's response was to paint *Goldfish*, an oneiric composition portraying a goldfish head and several nude female figures, one of whom appears to be 'mooning' the viewer. (Figure 2). Originally titled *To my detractors*, the message of the painting was obvious.

CREDIT: Heritage Image Partnership Ltd/Alamy Stock Photo.

The final painting in the University of Vienna series was *Jurisprudence* (completed in 1903), in which Klimt renews his instinctual approach and nude imagery, portraying an emotional critique of our competence to judge and punish other human beings.

The Ministry of Education ultimately ruled the paintings unworthy of the Great Hall of the University of Vienna. Despite strong resistance by the state, which owned the three paintings, Klimt was able to recover them with the help of friends. The paintings subsequently changed hands several times (Klimt died in 1918 during the influenza pandemic), and were later seized by the Nazis from their Jewish owners in 1938, following the annexation of Austria by Germany. Stored at the Schloss Immendorf Castle in lower Austria, the three ceiling paintings were ultimately destroyed in 1945 when retreating SS troops set the castle on fire. However, Goldfish survived and is now housed at the Solothurn Art Museum in Switzerland. Moreover, copies of it still have the potential to cause offence: in 2014 – 114 years after the initial criticism of Klimt's paintings – the then dean of the Albert Einstein College Medicine in New York, an institution renowned for its progressive tradition, asked me to remove a reproduction from the hallway outside my laboratory because he felt some people might consider images of naked women inappropriate for a medical school.

## Is there a relationship between art and science?

Could it be possible that Klimt, intuitively, had a more accurate understanding of the nature of medicine than some doctors and university administrators of the time? The understanding of medicine was certainly biased in the case of the latter, by their role and position in society, but that was not the case for Klimt. The writer Jorge Luis Borges once said that 90% of art wouldn't exist if we knew what follows death (Gelman, 2011), suggesting that art is a search for answers to questions about the meaning of our existence, which is not that different from what drives scientists in their quest for knowledge. Artists often combine many talents – painters can also be writers and musicians, and vice versa, indicating that their creative impulses can be expressed via multiple avenues. Likewise, Leonardo da Vinci was an example of someone who channel their creativity into both art and science.

Moreover, scientists sometimes think as artists when developing their scientific ideas. Indeed, Albert Einstein reportedly said that he "never thought in logical symbols or mathematical equations, but in images, feelings, and even musical architectures" (Root-Bernstein and Root-Bernstein, 2010; Wertheimer, 1945). And elsewhere Einstein referred to the relationship between art and science more formally and explicitly when he wrote: "After a certain high level of technical skill is achieved, science and art tend to coalesce in esthetics, plasticity, and form. The greatest scientists are always artists as well" (Calaprice, 2000).

Art and science are considered by many to be complementary forms of knowledge. But in what ways are they complementary? The poet does not give answers," the poet Juan Gelman wrote in 2011. Until the end of his days he interrogates the invisible reality, which does not give him answers" (Gelman, 2011). Einstein’s thoughts on art and science expanded this notion, noting: "If what is seen and experienced is portrayed in the language of logic, then it is science. If it is communicated through forms whose constructions are not accessible to the conscious mind but are recognized intuitively, then it is art." According to this view, Klimt had an intuitive understanding of medicine that more adequately described its real nature, an imperfect tool that mankind created to alleviate pain, rather than the grandiose voluntarist perspective of health professionals and university administrators of its time.

## Art, science and society

In contrast to other notable rejections of commissioned art, such as *The Conspiracy of Claudius Civilis* by Rembrandt (rejected by the city of Amsterdam) and *Man at the Crossroads* by Diego Rivera (plastered over at the Rockefeller Center), the fate of the University of Vienna ceiling paintings continue to interest scientists and scholars (Finn et al., 2013; Kandel, 2012; Sark, 2011; Schorske, 1981).

The findings of scientists and the intuitive insights of artists are often resisted by the public, as they generally question the religious and cultural beliefs that prevail at the time (see, for example, the *Prison Notebooks* of Antonio Gramsci). History is rich with examples of this conflict. The findings of Galileo were severely questioned by the Catholic church, and the *The Marriage of Figaro* by Mozart was banned in Austria for its anti-aristocratic and anti-militaristic nuances. And paintings by Kandinsky, Klee, Kirchner, Marc and other German expressionists were condemned as 'degenerate' by the Nazi regime, while the work of Einstein and Helmholtz dismissed as 'un-Germanic', (Buchwald, 2016; Stern, 1986).

While we often think these clashes belong to a past from which we have evolved, they actually recur. In 1823, in a play called *Almansor*, Heinrich Heine made a grim prediction (Where books are burned, in the end, people will also be burned") that, tragically, was fulfilled in Germany during the Nazi regime and in Chile during the 1973 military coup. Moreover, scientists and artists are often among the first to be jailed and prosecuted by dictatorial regimes. And Bertolt Brecht warned us to remain alert to the recurrence of the threats posed by dictators in the last sentence of his play *The Resistible Rise of Arturo Ui: A Gangster Spectacle*: "Do not rejoice in his defeat, you men. For though the world has stood up and stopped the bastard, the bitch that bore him is in heat again."

There are more recent examples of such tensions. In 1999 the then mayor of New York, Rudolph Giuliani, froze city funding to the Brooklyn Museum of Art and even threatened to evict it from its building because he found Chris Ofili’s painting *The Holy Virgin Mary*, portraying a black Madonna decorated with elephant dung, offensive to his sensitivity (Foggatt, 2018). Another, more extensive example, was destruction of historical religious buildings in Iraq and Syria by ISIS in 2014 and 2015.

An even more striking example of this tension, in my opinion, is the way that some governments deny climate change despite the overwhelming scientific evidence supporting it (Mellilo et al., 2014) and the catastrophic consequences it will entail for the planet. Worse still, the current administration in the United States is undoing the work of previous administrations and pulling out of international agreements. This episode is a clear example of how scientific progress does not necessarily guarantee social progress and how regressive policies can prevail despite common sense.

The human race is capable of generating great art and science, but it is also capable of opposing and destroying great art and science. We are immersed in an eternal fight between good and evil (or between creation versus destruction), a fight that Freud argued resides in the unconscious of every human being. As the sole survivor of the University of Vienna ceiling paintings by Klimt, *Goldfish* represents the resilience of art, as well as a defense of its essential contribution to human civilization.
